# Evaluation of Current and Former Teleradiology Systems in Africa: A Review

**DOI:** 10.5334/aogh.3711

**Published:** 2022-06-24

**Authors:** Brandon Ewing, David Holmes

**Affiliations:** 1 University at Buffalo Jacobs School of Medicine and Biomedical Sciences, Department of Family Medicine, 1001 Main Street Buffalo, NY 14203, US

**Keywords:** Teleradiology, Telemedicine, Africa, Telehealth, Radiology

## Abstract

**Background:**

Teleradiology has grown tremendously across the globe, providing significant benefits to both patients and physicians. In the late 1990s, South Africa sought to lead teleradiology adoption efforts by creating a national telemedicine system through a structured and phased approach. Although initial reports of the system’s effectiveness were encouraging, the present status of this project, as well as comparable efforts in surrounding developing countries, has remained uncertain.

**Objective:**

To explore the status of teleradiology adoption in Africa, identify existing barriers to adoption, and explore potential solutions to the most commonly identified barriers.

**Methods:**

A narrative literature review was conducted to find articles that discussed current and past teleradiology systems in Africa. Each item was evaluated for relevance separately based on specified inclusion and exclusion criteria and was used to field further articles if relevant to the topic, even if not found in the initial search. The search began with articles published after January 1995 and included articles through December 2021.

**Findings:**

Although teleradiology systems in Africa has shown to have a benefit in improving patient outcomes, current implementation remains limited due to feasibility projects with no singular picture archiving and communication system (PACS) being utilized at the time of writing.

**Conclusions:**

Although teleradiology has significant potential and can benefit the developing countries in Africa, further expansion, in terms of both complexity and adoption rates, remains hindered by infrastructure development, clinician and technologist support, and general sociopolitical factors.

## Introduction

Telemedicine is defined as the “delivery of healthcare and sharing of medical knowledge over a distance using telecommunication systems” [1]. Contrary to popular belief, the concept of telemedicine existed for much of the twentieth century. However, the modern concept of telemedicine did not surface until the second half of the twentieth century. Similar to any field, early attempts to bring the concept of telemedicine into practice were generally unsuccessful due to sheer technological limitations [1]. Telemedicine became more of a reality in the mid-to-late 1990s, when information and images could finally be saved digitally [1]. Nowadays, telemedicine is found to play a role in all fields of medicine. A common application of telemedicine is teleradiology. Teleradiology is often described as the transmission of radiological images from one location to another for interpretation or consultation. Furthermore, teleradiology systems are often different in structure. Most commonly, they serve to link the resources from high-income countries to low-income countries or serve as a link between central and satellite hospitals within a single country [2].

Since its inception, teleradiology has exploded in the United States and Europe. A 2019 survey conducted by the American College of Radiology estimated that nearly 86% of radiologists have performed teleradiology with the last ten years [3]. These numbers are similar to European countries, where radiologist engagement in teleradiology is estimated to be greater than 70% [4]. It is also estimated for the global teleradiology market revenue to grow to $8.2 billion by 2024.

The benefits of teleradiology, and telemedicine in general, have been well-described in the literature. Teleradiology has been demonstrated to be beneficial for both patients and physicians. A robust teleradiology program enables underserved communities to receive 24/7/365 high-end image interpretation that can help further their medical care without the need to travel long distances [5]. At academic centers, teleradiology has shown to be beneficial for helping to train radiologists, often broadening their knowledge and providing them with exposure to rare congenital and developmental diseases [6].

The surge of teleradiology in the United States and across the globe ignited interest in exploring its use in developing countries, such as those in Africa. Although initial developments in these countries were promising, the current status of teleradiology in Africa and elsewhere has not been well-defined in the literature. In 2010, it was estimated that nearly 75% of the world’s population has insufficient access to medical imaging [7]. This review will explore the current state and effectiveness of teleradiology in Africa, identify existing impediments to expansion and implementation, and discuss potential ways of overcoming these barriers.

## Methodology

A traditional literature review, using PubMed and Embase was conducted to explore the existing literature from 1995-2021 on the status of teleradiology in Africa, as well as any existing barriers to development.

The MESH terms and keywords were *Telemedicine, Teleradiology, Africa*. Each article was individually evaluated, and additional articles were included if relevant to the topic. The full list of search strings is listed in the Appendix. The search was conducted in December 2021. Literature inclusion and exclusion criteria are listed below.

**Inclusion Criteria**

- Published after 1995
- Relevant to global health AND Teleradiology OR health informatics
- Relevant to African communities
- Scientific, peer-reviewed studies OR governmental reports

**Exclusion Criteria**

- Not written in English
- Case reports
- Abstracts
- Full-text unavailable

## Results

### History of Teleradiology in Africa

Before the late 1990s, telemedicine and teleradiology developments in Africa were generally small and scattered pilot-type projects [8]. However, in 1998, the first major development in teleradiology occurred in South Africa at the Department of Health (DOH) [9]. The DOH sought to develop a National Telemedicine System in an attempt to increase primary health services in the rural parts of the country. To assist its effort, the DOH created a novel telemedicine task team to plan out the implementation strategy. The telemedicine task team consisted of public representatives from the Department of Health, Department of Communication, and representatives from Telkom SA, the national telecommunications company [9]. At the time, it was the most robust proposal for the implementation of teleradiology and telemedicine in general in Africa. Upon the development of the task team, working groups developed extensive guidelines, ranging from basic clinical protocols to security guidelines and code of ethics [9].

Implementation of the teleradiology network involved a phased approach, where an initial twenty-eight pilot sites were created across six provinces [9,10]. Each of these pilot sites was capable of teleradiology and several other medical disciplines [9,10]. Subsequent phases involved the expansion of the network dependent on relative demand. By the first year of implementation, a total of 2663 radiographic studies were performed, 264 of which were interpreted by a teleradiologist [9]. The remainder were explained directly by the primary care provider [9].

Initial reports on the status of implementation were positive. A 2002 follow-up report evaluating the project stated that preliminary feedback showed a “clear improvement in medical services” [11]. Perhaps the greatest benefit of the system was its ability to improve access to radiology specialist reporting in many rural parts of South Africa. In addition, the report claims that the system saw substantial improvements in the diagnostic and management skills of primary care physicians [11]. However, despite these claims, the report concludes by stating that the evaluation of the telemedicine system remains ongoing [11].

### Current status of teleradiology in Africa

Since the inception of the National Telemedicine System in South Africa, several other African countries have attempted to institute similar initiatives, including but not limited to Mauritania; Mozambique; Mali; Botswana, and Ethiopia [8]. Despite the broad range of expansion, the robustness of the development in each country remains unclear. Nevertheless, a careful review of the literature does identify some studies attempting to quantify the exact scope and success of teleradiology expansion. Table 1 provides a summary of the most prominent studies described below.

**Table 1:** Summary of research attempting to quantify the scope and success of teleradiology services in Africa.


STUDY	PUB YEAR	COUNTRY	STUDY DESIGN	RESULTS	BARRIERS IDENTIFIED

Coulborn et al. [17]	2012	Malawi	Descriptive analysis	One hundred fifty-nine images (from 158 patients) were reviewed by teleradiology. Teleradiology changed patient management in 36 cases (23.5%).	Image quality on teleradiology systems

Zennaro et al. [16]	2013	Angola	Feasibility study	Twenty thousand five hundred sixty-four digital X-ray images were created with no major technical problems and no need for on-site supervision. “Novel” digital radiology system retained and improved image quality.	Cost of equipment

Halton et al. [15]	2014	Multiple	Retrospective analysis	Mean teleradiologist response time is 6.1 hours. Seven out of eight respondents indicated teleradiologist consults found to be favorable; six out of six respondents indicated teleradiologist input to assist in the clarification of diagnosis.	Low and variable volume in the usage of teleradiology service

Sangare et al. [18]	2015	Mali	Retrospective analysis	Teleradiologists provided the sole diagnosis for 29% of cases. No diagnosis by regional physician decreased from 93% to 24%.	Infrastructure (internet connection); the low volume of cases; cost of services for patient and provider

Crumley et al. [19]	2020	Democratic Republic of Congo	Paired before-after study	Diagnosis changed following teleradiology in 62% of cases, and treatment plans changed in 61%.	Cost and maintenance equipment

Essop and Kekana [20]	2021	South Africa	Qualitative analysis	Narrative feedback predominately negative from referring clinicians and technologists.	Communication between consulting physician, teleradiologist, and technologist


Before 2010, the amount of literature regarding the exact scope of teleradiology systems in Africa was minimal. Very few original research articles were published. Of the original research articles published within this timeframe, many of them were either narrow in scope or were using teleradiology at its most basic level [12,13]. The most notable piece of literature during this timeframe was a study examining patient satisfaction with the IKON project, a startup teleradiology project in Mali. The IKON project sought to use teleradiology to connect radiology services from the smaller regional hospitals in Mali to the larger hospitals in Bamako, the nation’s capital [14]. The paper noted that at the time of writing, nearly all the radiologists in the country were located in the capital. Out of the 2 500 cases processed, the overall patient satisfaction with services was estimated to be 98% [14] In addition, it was further stated that the teleradiology services had saved the population more than 125 000 USD [14].

A retrospective analysis of radiology cases sent out for teleradiology consultation was conducted at about the same time by Médecins Sans Frontières (MSF) or “Doctors Without Borders”. At the time, MSF was perhaps one of the more robust teleradiology platforms, relying on the “store-and-forward” method for transmitting medical information, where images were sent on a secure website either through a DICOM (Digital Imaging and Communication in Medicine) or JPEG format [15]. The study found that across four years, the vast majority of teleradiology cases (470/564) from the MSF telemedicine system came from Africa, the majority of which (388/470) came from either Central African Republic or Malawi [15]. The relative satisfaction was also indicated by the fact that the majority of respondents (7/8) felt the radiologist’s advice useful, and all (6/6) reported that the service’s involvement helped clarify the diagnosis [15].

During data collection of the above study, a few other studies were published exploring the effectiveness of teleradiology in counties such as Malawi (2) and Angola (1). Although considered a feasibility study, the Angola study was unique for finding a way to implement a digital X-ray device in a low-resource setting [16]. In comparison, both Malawi studies found benefit in using teleradiology with one focusing on a decrease in no diagnosis and misdiagnosis rate while the other focusing on the benefits of teleradiology on screening for infectious diseases [17,18].

In 2015, a more robust study about the benefits of teleradiology in Africa was imitated with a particular focus on the Democratic Republic of the Congo (DRC). In collaboration with MSF and a local hospital in the DRC, researchers sought to conduct a retrospective paired before-after study on the therapeutic impact teleradiology had on the medical management of patients living in the DRC [19]. Similarly to the other studies stated, the final results were encouraging in that 62 percent of patients had their care plan changed [19]. Also, it found that the teleradiology system resulted in a notable decrease in the number of invasive interventions, such as orthopedic and interventional surgery, a conclusion not directly indicated in prior studies [19].

Most recently, a qualitative analysis of the South African teleradiology system was conducted by the University of Pretoria in South Africa in an attempt to define the extent of the county’s telemedicine system [20]. Of note, Pretoria was one of the first locations that adopted the strategy set forth by the National Telemedicine Task team in 1998 [9]. In this study, focus group discussions were held with radiographers and referring clinicians involved with teleradiology in the Northwest province of South Africa [20]. Contrary to other studies discussed, the narrative feedback was relatively negative. Several parties, especially the referring clinicians and technologists, felt inadequately supported and stated that they felt ill-equipped to fulfill their roles [20]. It is important to note that this study is not examining the direct benefits of teleradiology on communities, but rather the relative satisfaction with the system in place.

## Discussion

Based on the studies reviewed, it is apparent that the teleradiology system is not utilized to its fullest capacity, even though its potential is great. Nearly all studies published saw some sort of tangible benefit with teleradiology [14,15,16,17,18,19]. Upon examining the overall satisfaction, nearly all studies indicated that clinicians and patients generally had favorable opinions or documented benefits of teleradiology [14,15,17,19,20]. However, many of the studies identified a central problem in many of the teleradiology systems: lack of usage [15,17,19,20]. Halton et al. noted that although the user satisfaction with the teleradiologist input is favorable, there were significant fluctuations in demand, with some teleradiology sites sending fewer than 10 cases a month [15]. Furthermore, in the period investigated, Halton et al. asserted that the overall growth in the system has been zero-to-minimal [15].

Despite the relative success of several pilot projects, there are several barriers impacting expansion of teleradiology in Africa. These include problems with infrastructure, aptitude of providers/technologists, and underlying social/political support.

### Infrastructure

One of the most noted conclusions when examining the literature was the lack of volume in teleradiology cases. The most logical explanation for this is the lack of solid infrastructure for many developing countries in Africa [18]. Infrastructure is defined as the physical and organizational structures needed for an operation. In the context of teleradiology, infrastructure refers to elements such as computer and internet access or bandwidth [21]. When looking solely at internet access and usage, it varies widely in Africa with estimates ranging from 15% in rural areas to 50% in urban areas [22]. Fixed-broadband subscriptions, another criterion for analyzing internet availability, reveals similar results. According to a report from the International Telecommunication Union (ITU), there are 1 in 100 inhabitants in Africa who have fixed-broadband subscriptions. This is in contrast to 35 in 100 inhabitants in Europe and 23 per 100 inhabitants in the Americas [22]. It is plausible to believe that having unreliable infrastructure can impact provider trust in the system, thereby impacting demand. This might explain why several of the studies noticed low volume when attempting to examine the number of cases.

Correcting for infrastructure often creates additional issues as well. Some African countries have limitations on the type of medical equipment that can be imported. For example, in South Africa, the import of medical equipment is allowed, but an importer’s certificate is needed along with several other stipulations [23]. In contrast, Nigeria allows used medical equipment to be imported with zero duty charges [23]. As Desiraju et al. mentioned, the diffusion of technology in developing countries shares a positive correlation with national per capita spending [24]. Ultimately, without the proper infrastructure, it becomes increasingly challenging to adopt and sustain the technology that telemedicine requires.

As far as potential solutions for this matter, improving infrastructure is essential, but can take a significant amount of time to truly develop. Despite this, many smaller steps can be taken in the meantime and can provide incremental improvement for teleradiology systems. For example, some teleradiology systems have attempted to circumnavigate such infrastructure issues by having images scanned or photographed and sent to radiologists by email [25]. Some have even used JPEG compression to speed up the process; however, it is crucial to note that this can result in reduced image quality [5]. Additionally, this type of image modification might not be the best approach unless absolutely necessary, as study quality is already subject to how exactly a technologist performs a study and whether it follows a standardized approach [5,26].

Another potential area to explore is the utilization of mobile phones and networks as a way to circumnavigate issues with internet connectivity. In contrast to traditional broadband networks that operate over a wire, mobile networks provide a way to access the internet wirelessly. As Bagayoko et al. points out, attempts to investigate this topic are now underway and appear to be promising [14]. A feasibility study in November 2021 found that fifth-generation (5G) mobile networks have significant potential to transmit medical imaging data [27]. Being able to harness the power of these networks could perhaps accelerate the development of teleradiology in these low-income countries.

### Aptitude of providers and technologists

Another identifiable barrier from the literature was the lack of support and proper education of physicians in developing countries. Although only one article directly addressed this, it is plausible to assume that this barrier may have been a factor impacting the utilization rates of the teleradiology service. Essop and Kekana’s research provided a glimpse of such issues through narrative feedback. In the article, it is highlighted that a common frustration between teleradiologist, referring clinicians and technologists was a feeling of disconnect between one another [20]. In particular, each party is frequently asked to perform duties outside the scope of their job description. For example, when referring clinicians attempted to reach out to teleradiologists over concerns regarding authorization, the radiologist was often unwilling to engage with them [20]. On the flipside, radiographers often felt dismissed by referring clinicians whenever they offered their professional opinion [20].

Other articles have stated that the efficiency of any teleradiology system is contingent upon having a robust chain of command [5]. Without this, it is easy for the most ambitious projects to become unsustainable in the long term [16]. Upon inspection of the literature, it becomes clear that these same issues often impact telemedicine in general. A recent meta-analysis exploring the general barriers of telemedicine in Sub-Saharan Africa found similar issues. It found that both patients and professionals frequently lacked proper knowledge and training in telemedicine, which was especially evident in less developed countries such as Rwanda and Uganda [28]. Furthermore, it was suggested that this lack of expertise could potentially impact the adoption rate of said system [28].

Potential solutions for this issue were more crystalized when compared to the other barriers. It becomes abundantly clear that some sort of teleradiology-specific training is necessary to help bridge the gap between the parties involved. In South Africa, several telemedicine training programs seek to train technologists. However, these do not cater to teleradiology in particular [29]. Before these endeavors are explored, it is reasonable to say that more research needs to be conducted to ensure that the needs of a particular country are fully understood. In essence, the more individualized a teleradiology curriculum is to a country, the greater the likelihood it will be perceived as beneficial by its students. In addition, some have found ways to overcome education hurdles. For example, Imaging the World (ITW), an organization that prides itself on ensuring that low-income countries have access to medical knowledge, has a standardized protocol for ultrasound scanning using surface landmarks [5]. Such protocols could help improve efficiency when expertise is limited. This is especially relevant considering ultrasound is a technique that is heavily user dependent. Lastly, it is important to mention that radiologists are made aware of the challenges other parties face, as mutual understanding is vital to creating a robust system. Perhaps tele-training programs could be created to help circumnavigate such issues, where specialists in high-income countries become connected to physicians and technologists in low-income countries. Having these types of connections will not only help foster understanding but provide opportunities for life-long-learning and growth as technology advances.

### Underlying social and political support

Perhaps one of the most underrated barriers to successful teleradiology adoption within African countries is the respective socio-political system in which it operates. Although this was not directed examined in the cases discussed above, there is evidence to indicate that socio-political systems have a tremendous impact on the diffusion of technology [30]. As mentioned before, having a proper infrastructure is critical for having a successful telemedicine system. However, it must also be remembered that healthcare delivery is often proportional to prior financial support, which is often dictated by the government [28]. If a government does not support a particular initiative, it is unlikely that it will provide any sustainable financial support. This is evident when looking at the broader attempts to bring telemedicine to the forefront. For example, when looking at eHealth implementation efforts by the Botswanan government, it can be seen that on multiple occasions lofty goals were set across several without any additional planning or consideration [31]. These goals were never met, which is not surprising. In fact, as Ncube et al. noted that the only tangible framework for the system was in the form of an appendix to the government’s ICT policy [31].

Aside from the links between government, planning, and financial assistance, it is critical to the battle between innovation and culture. Many African countries have an emphasis on the elder, whereas new ideas mostly come from the person that is most senior in a department [31]. As Nucube et al. stated, it is common for new ideas to quickly fall out of favor if this is not considered [31]. Although this concept may seem self-centered to outsiders, it is important to remember that resources are limited, and teleradiology is often competing against other equally important public health measures, such as disease control or maintaining a clean water supply [19]. Although this concept is perhaps more ambiguous than others, understanding the culture and core tenants is vital whenever implementing a new idea, especially as an outsider.

Addressing this particular barrier remains difficult. As seen with many of the studies, groups such as Medicine without Borders can help mitigate some of the financial barriers that might be created by non-modifiable socio-political factors. It is critical that the benefits of such projects are not just communicated to governments but are also tied to tangible health benefits. For example, Nucube el al. found a significant problem with the draft of the Botswana eHealth Strategy [31]. Although not specifically related to teleradiology, it highlights an important point: the general “informatics tone” did not have a clear appeal to those that it was being marketed toward [31]. It is reasonable to believe that the lack of a palpable appeal was the main cause of the project’s stalling. Once again, this underscores the importance of having a good strategy that clearly delineates the goals of a project and the potential benefits to the community.

Another potential solution not mentioned in the studies discussed above is exploring the use of hospital partnerships between high and low-income countries. As observed by Chetwood et al., this solution already exists in the context of biomedical research [32]. There have been many instances of international partnerships that have sought to bring research funding, and thereby medical support, to low-income countries [32]. However, as noted by the authors, these partnerships must remain purposeful and practical. It is common for the ultimate outcomes of these partnerships to favor the high-income country [33,34]. Ultimately, without political and cultural support for such initiatives, it remains incredibly challenging to sustain such an implementation on a large scale.

#### Summary of recommendations to address barriers

*Infrastructure*

- Utilize JPEG compression to transmit images in places with poor internet connectivity.
- Shape teleradiology services around mobile networks in places where broadband connectivity is limited.

*Aptitude of Providers and Technologists*

- Conduct further studies to assess country-specific needs for teleradiology.
- Build teleradiology-specific curriculum for technicians in low-income countries with the utilization of standardized techniques for obtaining images.
- Develop and implement tele-training programs between experts and those who want to be trained in specific areas related to radiology.

*Underlying Social and Political Support*

- Work with local governments to establish concrete and specific objectives for teleradiology implementation in low-income countries.
- Connect universities and private practice radiology groups in high-income countries with hospitals in low-income countries.

As this is a review, the ability to critically appraise the literature remains dependent on the availability of studies in the context of the search criteria. As stated, in Table 1, much of the studies conducted were either retrospective or feasibility studies. Although valuable contributions to the literature, these were not randomized control trials, which creates the potential for bias. In addition, since this review was primarily narrative in nature, the results are unable to be quantified systematically. Nevertheless, considering the scarcity of review articles on this topic, a narrative review of this topic is still beneficial as it sheds light on the status of teleradiology in Africa and the barriers to expand these important services in Africa.

## Conclusion

This review has examined the current status of teleradiology in Africa, identified existing barriers to implementation, and explored potential solutions to these barriers. Despite several relatively successful pilot projects, teleradiology efforts remain scattered with minimal efforts to expand. As discussed, there are suggestions that can alleviate the problem, such as studying a country’s specific needs for teleradiology and exploring the use of mobile networks in places with low broadband connectivity. Finally, sustainable growth necessitates enhanced infrastructure, training, working relationships between teleradiologists, technicians, and referring doctors, as well as improved governmental supervision and coordination.
